# Mice with Whole-Body Disruption of AMPK-Glycogen Binding Have Increased Adiposity, Reduced Fat Oxidation and Altered Tissue Glycogen Dynamics

**DOI:** 10.3390/ijms22179616

**Published:** 2021-09-05

**Authors:** Natalie R. Janzen, Jamie Whitfield, Lisa Murray-Segal, Bruce E. Kemp, John A. Hawley, Nolan J. Hoffman

**Affiliations:** 1 Exercise and Nutrition Research Program, Mary MacKillop Institute for Health Research, Australian Catholic University, Level 5, 215 Spring Street, Melbourne, VIC 3000, Australia; natalie.janzen@acu.edu.au (N.R.J.); jamie.whitfield@acu.edu.au (J.W.); bkemp@svi.edu.au (B.E.K.); john.hawley@acu.edu.au (J.A.H.); 2 St. Vincent’s Institute of Medical Research, Department of Medicine, University of Melbourne, 9 Princes Street, Fitzroy, VIC 3065, Australia; lmsegal@svi.edu.au

**Keywords:** AMP-activated protein kinase, carbohydrate-binding module, glucose homeostasis, metabolism, liver, skeletal muscle

## Abstract

The AMP-activated protein kinase (AMPK), a central regulator of cellular energy balance and metabolism, binds glycogen via its β subunit. However, the physiological effects of disrupting AMPK-glycogen interactions remain incompletely understood. To chronically disrupt AMPK-glycogen binding, AMPK β double knock-in (DKI) mice were generated with mutations in residues critical for glycogen binding in both the β1 (W100A) and β2 (W98A) subunit isoforms. We examined the effects of this DKI mutation on whole-body substrate utilization, glucose homeostasis, and tissue glycogen dynamics. Body composition, metabolic caging, glucose and insulin tolerance, serum hormone and lipid profiles, and tissue glycogen and protein content were analyzed in chow-fed male DKI and age-matched wild-type (WT) mice. DKI mice displayed increased whole-body fat mass and glucose intolerance associated with reduced fat oxidation relative to WT. DKI mice had reduced liver glycogen content in the fed state concomitant with increased utilization and no repletion of skeletal muscle glycogen in response to fasting and refeeding, respectively, despite similar glycogen-associated protein content relative to WT. DKI liver and skeletal muscle displayed reductions in AMPK protein content versus WT. These findings identify phenotypic effects of the AMPK DKI mutation on whole-body metabolism and tissue AMPK content and glycogen dynamics.

## 1. Introduction

The AMP-activated protein kinase (AMPK) is a central regulator of cellular energy balance and metabolism. AMPK becomes activated in response to reductions in cellular energy availability such as those induced by fasting and exercise, which are characterized by increases in ADP and AMP concentrations relative to ATP. When activated, AMPK stimulates several catabolic pathways to promote energy production and inhibits anabolic processes to preserve cellular energy homeostasis [1]. These downstream AMPK-regulated pathways facilitate increases in lipid oxidation, reductions in lipid synthesis, and stimulation of insulin-independent glucose uptake in skeletal muscle to help maintain cellular energy stores [2].

The AMPK αβγ heterotrimer contains a regulatory β subunit that binds the catalytic α and regulatory γ subunits [3]. The AMPK β subunit exists in two isoforms; in rodents, the β1 isoform is predominantly expressed in liver, while the β2 isoform is primarily expressed in skeletal muscle [4]. Both AMPK β subunit isoforms possess a carbohydrate-binding module (CBM) that allows AMPK to bind glycogen [4,5,6,7,8], the primary storage form of glucose in liver and skeletal muscle. In the two decades since AMPK’s glycogen-binding capacity was discovered, investigations directly examining the roles of AMPK-glycogen interactions have primarily been limited to in vitro studies. Glycogen was reported to have an inhibitory effect on AMPK activity in vitro [6], but we [7] and others [9] have not detected glycogen-dependent inhibition of AMPK in vitro. Experiments in rat skeletal muscle with reduced glycogen concentration have demonstrated a greater increase in AMPK activity in response to 5-aminoimidazole-4-carboxamide ribonucleotide (AICAR) administration compared to glycogen-replete skeletal muscle [10]. Similarly, human studies have demonstrated greater AMPK phosphorylation and kinase activity when exercise is commenced with low compared to normal or elevated skeletal muscle glycogen availability [11,12,13]. While these investigations have helped establish the hypothesis that glycogen binding may regulate AMPK activity and allow AMPK to “sense” existing energy stores [6], the physiological implications of this relationship cannot be fully elucidated by acutely modifying glycogen content in vivo (i.e., with exercise and muscle contraction) or examining AMPK and glycogen interactions in isolation in vitro. in vivo studies investigating the physiological consequences of disrupting AMPK-glycogen interactions are therefore warranted.

We previously generated two isoform-specific AMPK β knock-in (KI) mouse models in which tryptophan residues critical for AMPK-glycogen binding were mutated in either the β1 (W100A) or β2 (W98A) subunit isoform [14]. We demonstrated that mice with a KI mutation to chronically disrupt glycogen-binding capacity in a single AMPK β subunit isoform predominately expressed in liver or skeletal muscle displayed reduced total AMPK content and kinase activity, which was associated with increased fat content in liver and skeletal muscle of β1 W100A and β2 W98A KI mice, respectively [14]. Additionally, β2 W98A KI mice displayed increased whole-body fat mass and impaired glucose handling [14]. However, these KI mouse models targeted a specific β1 or β2 isoform with predominant tissue expression in either liver or skeletal muscle, respectively. Most rodent tissues, including liver and skeletal muscle, express detectable levels of both isoforms [4]. As a result, the phenotypic effects of simultaneous β subunit isoform KI mutations on whole-body and tissue metabolism in vivo remain unknown. Furthermore, the consequences of chronically disrupting AMPK-glycogen binding via β subunit KI mutations have not been tested in physiological settings of energy stress and energy excess (i.e., fasting and refeeding). To investigate these phenotypic effects and the potential for compensatory and/or synergistic consequences of KI of both AMPK β subunit isoforms, we crossed these single β1 W100A KI and β2 W98A KI mouse lines to generate AMPK β1/β2 double knock-in (DKI) mice. We hypothesized that relative to wild-type (WT) mice, DKI mice would display increased whole-body fat mass, glucose intolerance, and impaired tissue glycogen utilization and synthesis.

## 2. Results

### 2.1. DKI Mice Display Increased Body Mass Associated with Increased Adiposity

**Figure 1:**
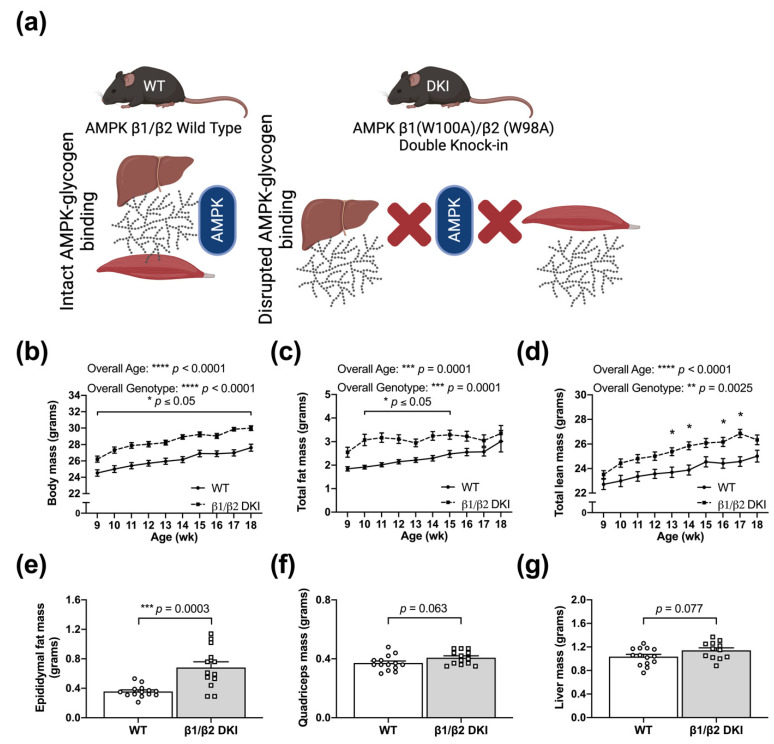
The double knock-in (DKI) mutation utilized to disrupt whole-body AMP-activated protein kinase (AMPK)-glycogen binding is associated with increased body mass and adiposity. (**a**) Schematic of AMPK wild type (WT) versus DKI mouse model in which tryptophan residues critical for AMPK-glycogen binding within the AMPK β1 and β2 subunit isoforms (predominantly expressed in the primary glycogen storing tissues liver and skeletal muscle, respectively) were mutated to alanine, resulting in chronic whole-body disruption of AMPK-glycogen binding. (**b**) Growth was assessed in male DKI and WT mice fed a standard rodent chow diet. EchoMRI analyses revealed that DKI mice had (**c**) greater total fat mass between the ages of 10 and 15 wk and (**d**) increased total lean mass between ages 13 and 17 wk, with the exception of 15 wk. Fed male mice, *n* = 11–30, 9–18 wk. Mice were culled and (**e**) epididymal fat pads, (**f**) quadriceps muscle, (**g**) and liver were excised and weighed. Fed male mice, *n* = 11–14, 17–20 wk. * *p* < 0.05, ** *p* < 0.01, *** *p* < 0.001, **** *p* < 0.0001.

To first determine how the DKI mutation utilized to chronically disrupt whole-body AMPK-glycogen binding influences body mass and composition, growth was assessed in age-matched WT and AMPK DKI male mice (Figure 1a) beginning at 9 wk of age. DKI mice displayed increased body mass compared to age-matched WT controls at all ages (Figure 1b). Increases in total fat mass and total lean mass were observed in DKI mice between 10–15 and 13–17 wk of age, respectively, versus WT mice (Figure 1c,d). Consistent with the observed increase in total fat mass, DKI mice also exhibited greater epididymal fat pad mass (Figure 1e). There were modest increases in the mass of glycogen-storing tissues, including skeletal muscle (quadriceps; *p* = 0.063; Figure 1f) and liver (*p* = 0.077; Figure 1g), but these were not statistically significant. Finally, DKI mice displayed greater pancreatic tissue mass relative to WT, while tissue mass of heart, gastrocnemius muscle, and soleus muscle were similar between genotypes (Table 1).

### 2.2. DKI Mice Have Reduced Voluntary Physical Activity, Increased Rates of CHO Oxidation, and Reduced Rates of Fat Oxidation

**Figure 2:**
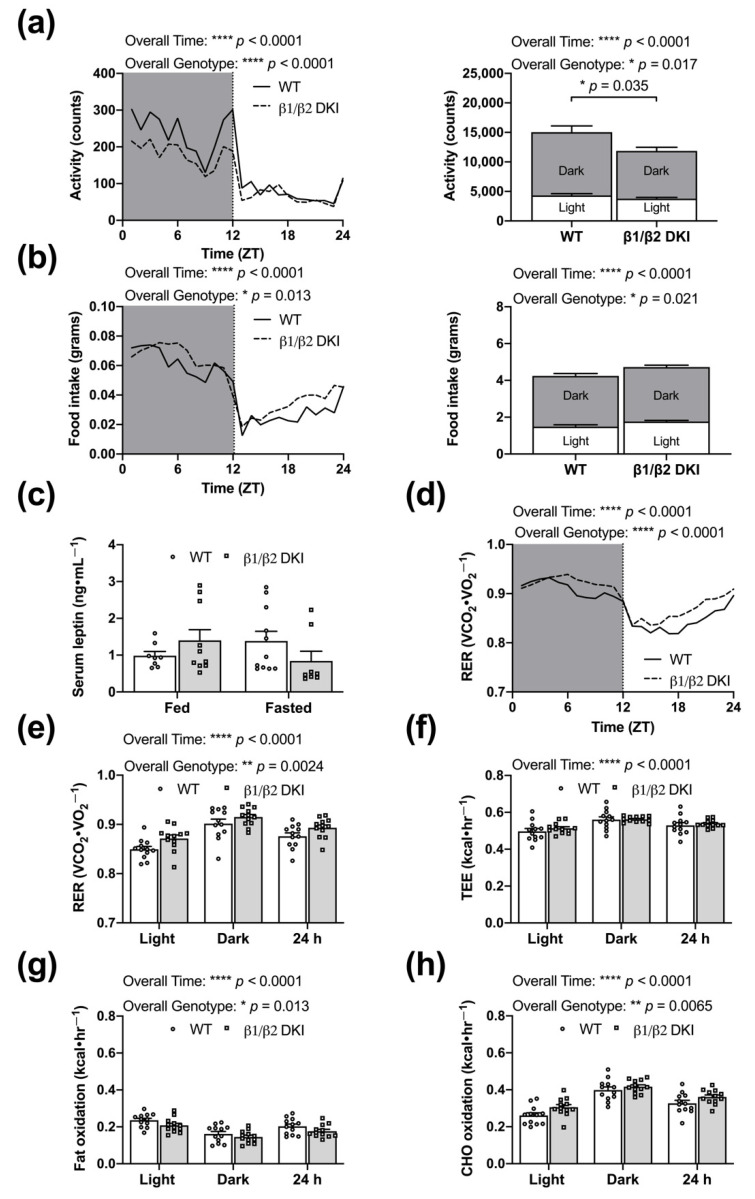
DKI mice have reduced voluntary activity and reduced rates of fat oxidation. Male DKI mice and age-matched WT controls were housed in metabolic cages and activity, food intake, oxygen consumption (VO_2_), and carbon dioxide production (VCO_2_) were measured every 18 min across ~60 h (after initial ~6 h acclimatization period). Activity data represent the total beam breaks in the x-ambulatory field. Hourly, 12 h light and dark phases (Zeitgeber time; ZT), and 24 h averages were calculated for activity and food intake. (**a** and **inset**) DKI had lower ambulatory activity, specifically during the dark phase, and (**b** and **inset**) a main effect for increased food intake, but no significant differences in either the light or dark cycles. (**c**) The main effect of increased food consumption was not associated with changes in serum leptin concentration in the fed or 6 h fasted states. (**d**,**e**) Respiratory exchange ratio (RER) was calculated and averaged for hourly, phase, and 24 h measures. (**f**) Total energy expenditure (TEE), (**g**) rates of fat oxidation, and (**h**) rates of carbohydrate (CHO) oxidation were calculated and averaged across the light, dark, and 24 h cycle. Fed male mice, *n* = 12, 12–16 wk. * *p* < 0.05, ** *p* < 0.01, **** *p* < 0.0001.

To assess potential whole-body metabolic changes underlying the observed differences in body mass and composition, DKI and WT mice were subjected to metabolic cage analysis. Changes in body mass and composition were associated with a main effect for decreased voluntary ambulatory activity in DKI mice versus WT, with significant genotype differences during the dark phase (Figure 2a and inset). There was a main effect for increased food intake in DKI mice, although post hoc analysis did not identify any genotypic differences during either the light or dark phases (Figure 2b and inset). There were no associated differences in serum leptin concentration in either the fed or 6 h fasted state compared to WT (Figure 2c). While there was an overall genotype effect of an increased respiratory exchange ratio (RER) in DKI mice versus WT (Figure 2d), individual differences in the light phase, dark phase, or 24 h period failed to reach statistical significance (Figure 2e). Furthermore, there were no differences in total energy expenditure (TEE; Figure 2f), despite overall genotype effects of decreased rates of fat oxidation (Figure 2g) and increased carbohydrate (CHO) oxidation (Figure 2h) in DKI mice versus WT. Consistent with the RER data, individual differences in overall substrate oxidation during light and dark phases or across 24 h did not reach statistical significance (Figure 2g,h). 

### 2.3. DKI Mice Display Hyperinsulinemia and Glucose Intolerance but Normal Whole-Body Insulin Sensitivity

**Figure 3:**
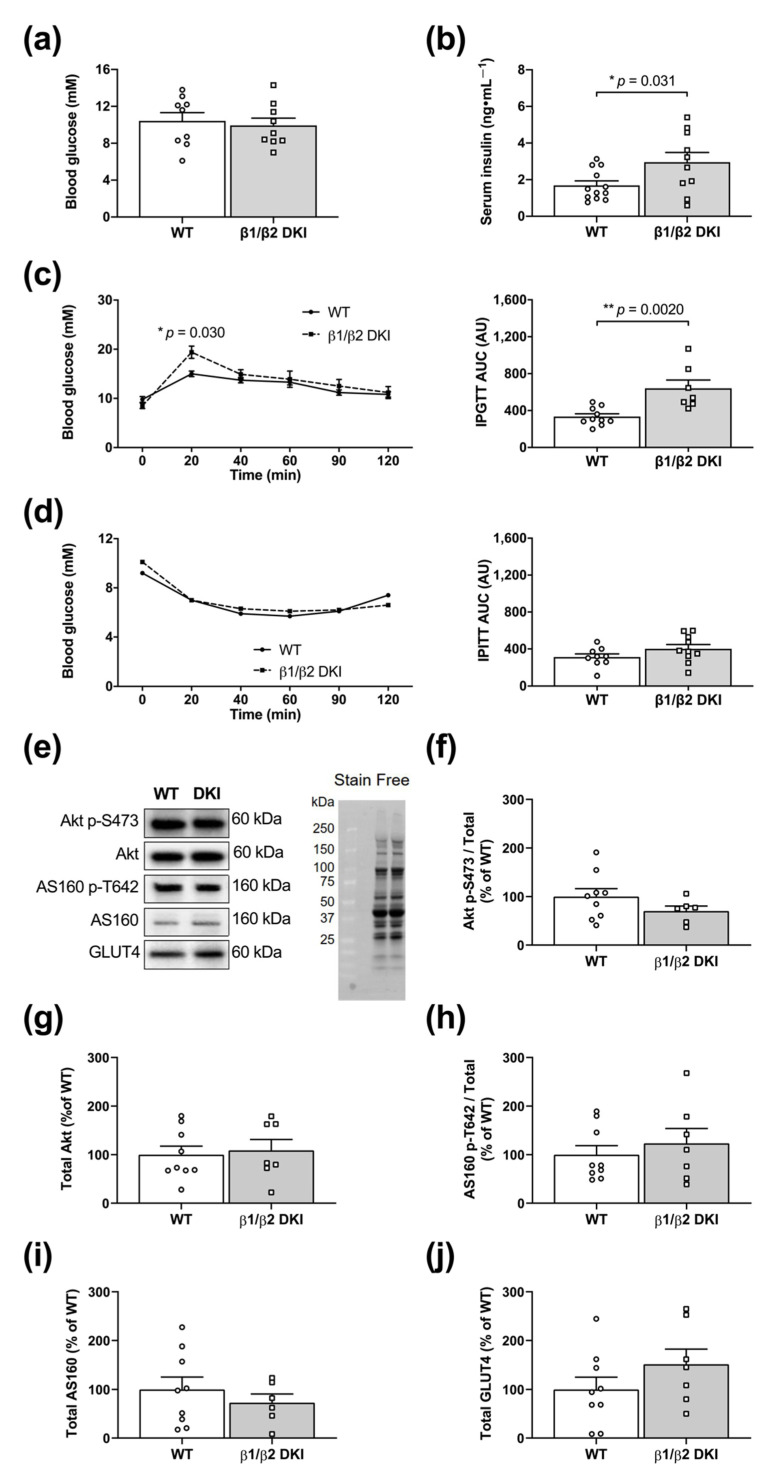
DKI mice are glucose intolerant but maintain whole-body insulin sensitivity. (**a**) Blood glucose was assessed via tail tip bleed in male DKI and WT mice following a 6 h fast. Male mice, *n* = 9, 17–20 wk. (**b**) Blood was collected via retro-orbital bleed from DKI and WT mice following a 6 h fast, and serum samples were analyzed for insulin concentration. Male mice, *n* = 10–12, 20–27 wk. (**c** and **inset**) Following a 6 h fast, DKI and WT mice were injected intraperitoneally with glucose (1 g·kg^−1^ total lean mass), and blood glucose responses were measured over 2 h post-injection and area under the curve (AUC), with baseline as the mean blood glucose at time 0 for each genotype, was calculated to assess glucose handling. Male mice, *n* = 7–10, 17–20 wk. (**d** and **inset**) Following a 4 h fast, DKI and WT mice were injected intraperitoneally with insulin (0.5 units·kg^−1^ total body mass), and blood glucose responses were measured over 2 h and AUC was calculated. Male mice, *n* = 9–10, 17–20 wk. Skeletal muscle was collected from male DKI and WT mice in the fed state, and phosphorylation status and total content of proteins associated with skeletal muscle glucose transport were quantified. (**e**) Representative immunoblots and stain-free image and quantified results of (**f**) Akt p-S573, (**g**) total Akt, (**h**) AS160 p-T642, (**i**) total AS160, and (**j**) total GLUT4 content in DKI versus WT skeletal muscle. Protein quantification was performed by normalizing the band intensity of the protein of interest to total lane protein using stain-free imaging, and phosphorylation was expressed relative to respective total protein content. Fed male mice, *n* = 7–9, 20–32 wk. * *p* < 0.05, ** *p* < 0.01.

To determine the phenotypic effects of the DKI mutation on whole-body glucose homeostasis, a series of blood and serum analyses were performed. Blood glucose concentrations were similar between DKI and WT mice in the 6 h fasted state (Figure 3a); however, 6 h fasted DKI mice displayed increased serum insulin levels compared to WT (Figure 3b). DKI mice subjected to intraperitoneal (IP) glucose tolerance testing (IPGTT) displayed increased blood glucose levels 20 min after glucose injection, associated with an elevated blood glucose area under the curve (AUC) compared to WT (Figure 3c and inset). However, there were no differences in blood glucose responses nor AUC between genotypes during IP insulin tolerance testing (IPITT; Figure 3d and inset). Tissue analyses were next performed to assess total protein content and phosphorylation status of glucose transport-related proteins in skeletal muscle (Figure 3e). There were no differences in Akt S473 phosphorylation status and total Akt protein content between DKI and WT skeletal muscle in the fed state (Figure 3f,g), nor changes in T642 phosphorylation and total protein content of its downstream target Akt substrate of 160 kDa (AS160; Figure 3h,i). Skeletal muscle GLUT4 protein content was also similar between genotypes (Figure 3j).

### 2.4. DKI Mice Have Decreased Voluntary Ambulatory Activity and Decreased Rates of Fat Oxidation during Overnight Fasting

**Figure 4:**
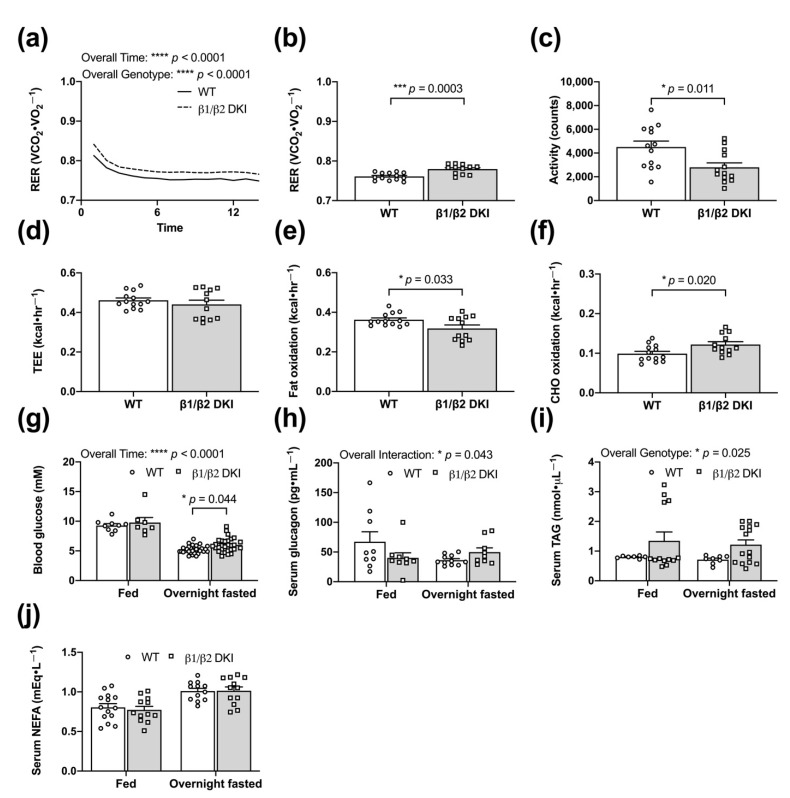
DKI mice have decreased voluntary activity and decreased rates of fat oxidation during overnight fasting (~14 h). Male DKI mice and age-match WT controls were singly housed in metabolic cages and acclimatized for ~6 h. At the beginning of the dark cycle (1800 h), food was removed from cages and respiratory gases and ambulatory activity were measured every 18 min during the overnight fast (~14 h). (**a**) Average hourly RER following induction of fasting. (**b**) Average RER. (**c**) Activity data represented by the total beam breaks in the x-ambulatory field during the fasting period. (**d**) TEE, (**e**) rates of fat oxidation, and (**f**) rates of CHO oxidation were calculated and averaged across the fasting period. Fasting male mice, *n* = 12–14, 17–20 wk. (**g**) Blood glucose was assessed via tail tip bleed in the fed (*n* = 7–9) and overnight fasted (*n* = 29–30) states. Overnight fasted mice in panel (**g**) consists of the three cohorts (overnight fasted, 5 h glucose, and 24 refed) described in Figure 5. Male mice, 20–32 wk. Blood was collected via retro-orbital bleed following overnight (~14 h) fast and serum samples were analyzed for (**h**) glucagon (*n* = 9–11), (**i**) triglyceride (TAG; *n* = 7–15), and (**j**) non-esterified fatty acid (NEFA; *n* = 12–14) concentrations. Male mice, 17–28 wk. * *p* < 0.05, *** *p* < 0.001, **** *p* < 0.0001.

To determine whole-body responses of DKI mice to acute energy stress relative to WT, mice were overnight fasted (~14 h) in metabolic cages. An overall genotype effect was detected for an increased RER in DKI mice compared to WT across the fasting period (Figure 4a), consistent with an observed increase in average RER (Figure 4b). DKI mice had reduced total ambulatory activity compared to WT (Figure 4c); although there were no differences in TEE between genotypes (Figure 4d). Consistent with overall genotype effects observed in the fed state, DKI mice displayed a decrease in rates of fat oxidation (Figure 4e) with a reciprocal increase in rates of CHO oxidation (Figure 4f). While no differences in blood glucose concentration were observed between genotypes in the fed state, there was a greater reduction in blood glucose levels following the overnight fast in WT compared to DKI mice (Figure 4g). Despite an overall interaction effect of genotype and condition for serum glucagon levels, there were no significant differences observed in WT versus DKI mice in either the fed or overnight fasted states (Figure 4h). Serum analyses to assess circulating fat availability revealed a genotype effect for increased serum triglyceride (TAG) concentration in DKI mice compared to WT; however, post hoc analysis revealed no significant individual differences in either the fed or overnight fasted states (Figure 4i). WT and DKI mice had similar serum non-esterified fatty acid (NEFA) concentrations in the fed state, and NEFA levels increased to a similar extent in response to an overnight fast in both genotypes (Figure 4j). 

### 2.5. DKI Mice Display Reduced Liver Glycogen Concentration in the Fed State and Altered Fasting-Induced Skeletal Muscle Glycogen Depletion

**Figure 5:**
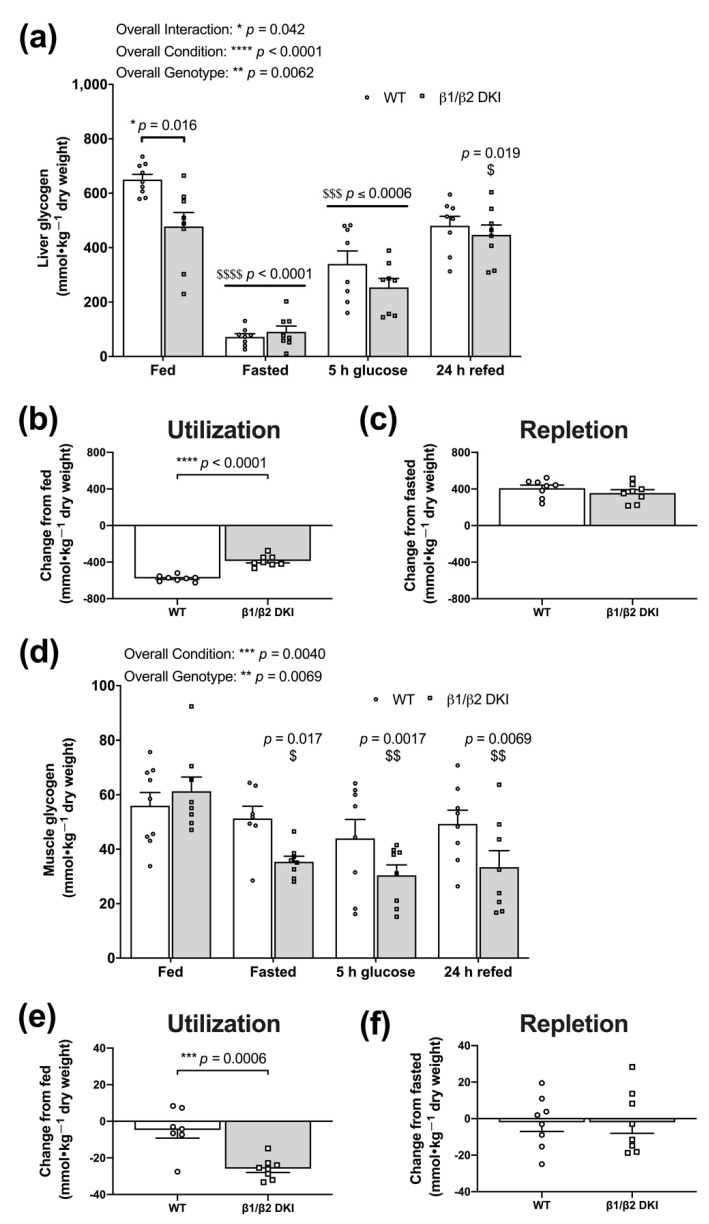
DKI mice display reduced hepatic glycogen concentration in the fed state and increased skeletal muscle glycogen utilization in response to fasting. Liver and skeletal muscle were collected and snap-frozen from male mice in the fed and overnight fasted states, and 5 h- and 24 h-post-administration of a glucose bolus (3.6 g·kg^−1^ total body mass) via oral gavage. The 24 h-post-cohort had *ad libitum* access to chow beginning at 5 h-post-glucose gavage. (**a**) Liver glycogen concentration was assessed in the fed state, overnight fasted state, 5 h-post-glucose gavage, and 24 h-post-glucose gavage and refeeding. (**b**) Utilization was measured as the difference in total glycogen content between fed and overnight fasted conditions. (**c**) Repletion was measured as the difference in total glycogen content between overnight fasted and 24 h-post-glucose gavage and refeeding. (**d**) Fed skeletal muscle glycogen levels were similar between DKI and WT mice but were only significantly reduced in DKI mice following an overnight fast. Glycogen remained suppressed below fed levels at both 5 h-post-glucose gavage and 24 h-post-glucose gavage and refeeding in DKI mice. (**e**) DKI mice had significantly increased utilization of skeletal muscle glycogen compared to WT when subjected to an overnight fast. (**f**) There was no significant glycogen repletion observed in either genotype from the overnight fasted state to 24 h-post-glucose gavage and refeeding. Due to the large number of mice needed for these experiments, a larger range of mouse ages (20–32 wk) was used, *n* = 7–9. Brackets represent comparison between genotypes within a condition and horizontal bars represent comparison with the fed condition for both genotypes. $ *p* < 0.05, $$ *p* < 0.01, $$$ *p* < 0.001, $$$$ *p* < 0.0001 versus fed condition from same genotype; * *p* < 0.05, ** *p* < 0.01, *** *p* < 0.001, **** *p* < 0.0001.

Based on genotypic differences observed in patterns of whole-body substrate utilization in both the fed and fasting states, we next investigated the phenotypic effects of the DKI mutation on the physiological regulation of tissue glycogen stores. DKI mice displayed reduced liver glycogen content in the fed state compared to WT (Figure 5a). Following an overnight fast, liver glycogen levels were depleted to similar levels in both DKI and WT mice; however, the net difference in liver glycogen from the fed to fasted state was 33% lower in DKI mice because of their lower glycogen content in the fed state (Figure 5b). To assess glycogen repletion, mice received an oral glucose gavage following an overnight fast. Liver glycogen content did not completely return to fed “baseline” levels 5 h-post-glucose gavage in either genotype but was restored to fed levels in DKI mice by 24 h-post-glucose gavage and refeeding (Figure 5a). However, WT and DKI mice displayed similar levels of total glycogen repletion from the overnight fasted state to 24 h-post-glucose gavage and refeeding (Figure 5c).

In skeletal muscle, glycogen content remained unchanged in WT following the overnight fast (Figure 5d,e). Meanwhile, there was a significant reduction in glycogen content observed in DKI mice following overnight fasting (Figure 5d), resulting in ~4-fold greater utilization of skeletal muscle glycogen from the fed to fasted state in DKI mice compared to WT (Figure 5e). Given skeletal muscle glycogen content was not altered in WT mice following the overnight fast, there was little estimated repletion 24 h-post-glucose gavage and refeeding (Figure 5f). However, in contrast to liver, skeletal muscle glycogen levels in DKI mice remained suppressed relative to baseline even 24 h-post-glucose gavage and refeeding (Figure 5d), resulting in negligible glycogen repletion (Figure 5f).

### 2.6. Liver and Muscle from DKI Mice Display No Changes in Glycogen Associated Proteins Relative to WT

**Figure 6:**
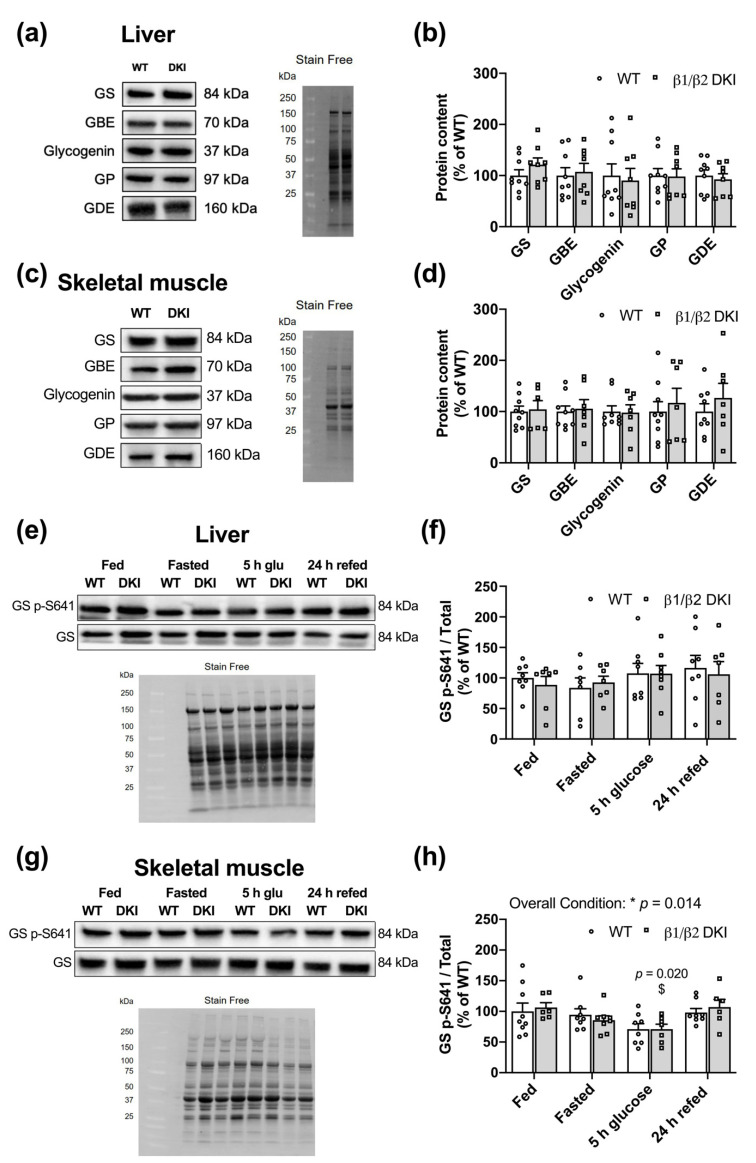
Liver and skeletal muscle from DKI mice display no alterations in content of glycogen-associated proteins. Tissues were collected from fed male mice and assessed for glycogen-associated protein content and phosphorylation status. (**a**) Representative immunoblots and stain-free image for DKI versus WT liver. (**b**) Quantified glycogen synthase (GS), glycogen branching enzyme (GBE), glycogenin, glycogen phosphorylase (GP), and glycogen debranching enzyme (GDE) in liver. (**c**) Representative immunoblots and stain-free image for DKI versus WT skeletal muscle. (**d**) Quantified GS, GBE, glycogenin, GP, and GDE in skeletal muscle. To assess phosphorylation of GS S641, liver and skeletal muscle were collected from male mice in the fed and overnight fasted states, and 5 h- and 24 h-post-administration of a glucose bolus (3.6 g·kg^−1^ total body mass) via oral gavage. The 24 h-post cohort had *ad libitum* access to chow beginning at 5 h-post glucose gavage. Representative immunoblots and stain-free images for DKI versus WT (**e**) liver and (**g**) skeletal muscle. Quantified GS p-S641 relative to total protein in (**f**) liver and (**h**) skeletal muscle. Fed male mice, *n* = 7–9, 20–32 wk. $ *p* < 0.05 versus fed condition from same genotype;* *p* < 0.05.

To determine if the observed differences in tissue glycogen content were due to changes in proteins associated with the regulation of glycogen breakdown and/or synthesis, glycogen-associated proteins were assessed in liver (Figure 6a) and skeletal muscle (Figure 6c). Changes in tissue glycogen dynamics observed in fed DKI mice were not accompanied by any differences in total glycogen synthase (GS), glycogen branching enzyme (GBE), glycogenin, glycogen phosphorylase (GP), or glycogen debranching enzyme (GDE) in either liver (Figure 6b) or skeletal muscle (Figure 6d). We next measured the phosphorylation status of GS, one of the rate-limiting enzymes in glycogen synthesis, in liver (Figure 6e) and skeletal muscle (Figure 6g) in the fed, overnight fasted, 5 h-post-glucose gavage, and 24 h-post-glucose gavage and refeeding states. There were no differences between genotypes nor across conditions observed for phosphorylation of GS S641 in liver (Figure 6f). There was an overall condition effect observed for phosphorylation of GS S641 in skeletal muscle; however, post hoc analysis did not reveal individual differences between WT and DKI skeletal muscle across conditions (Figure 6h).

### 2.7. DKI Mice Have Intact AMPK and ACC Phosphorylation in Liver and Skeletal Muscle

**Figure 7:**
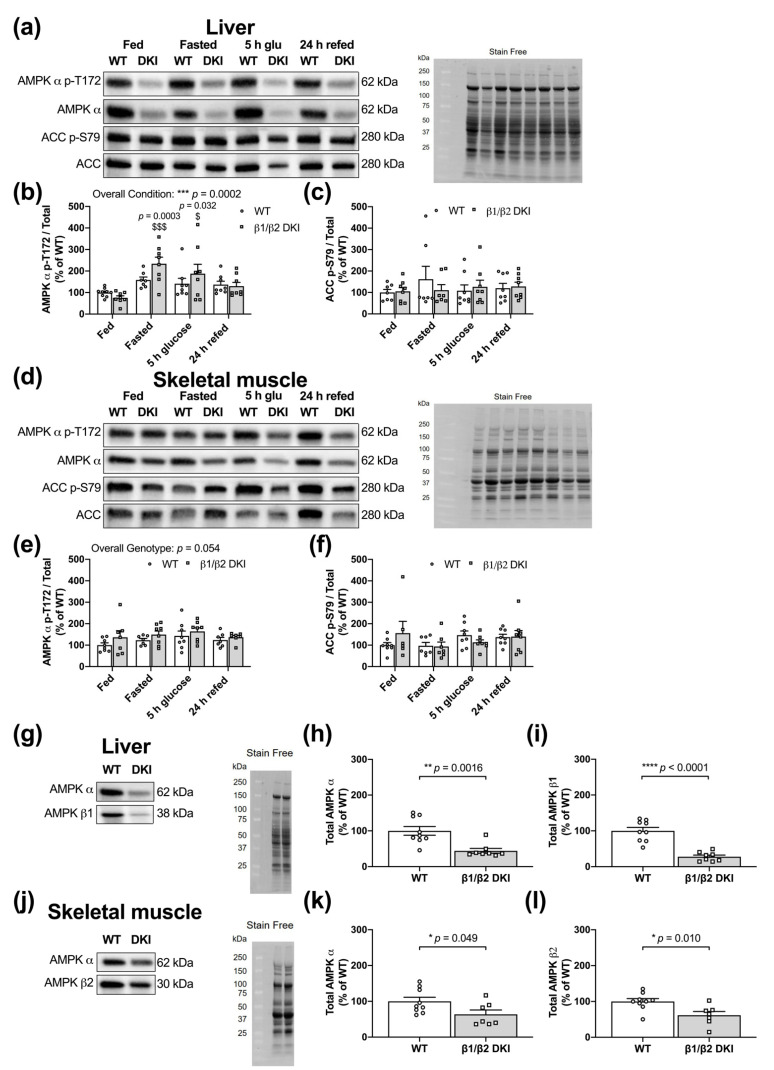
DKI mice have intact AMPK-acetyl-CoA carboxylase (ACC) signaling but reduced AMPK content in liver and skeletal muscle. Tissues were collected from male mice in the fed and overnight fasted states, and 5 h- and 24 h-post-administration of a glucose bolus (3.6 g·kg^−1^ total body mass) via oral gavage. The 24 h-post cohort had *ad libitum* access to chow beginning at 5 h-post glucose gavage. Liver and skeletal muscle were collected, snap-frozen and phosphorylation status of AMPK T172 and ACC S79 was assessed. Representative immunoblots and stain-free images for DKI versus WT in (**a**) liver and (**d**) skeletal muscle. Quantified (**b**) AMPK p-T172 and (**c**) ACC p-S79 relative to respective total protein in liver. Quantified (**e**) AMPK p-T172, (**f**) ACC p-S79 relative to respective total protein in skeletal muscle. Given the predominant glycogen stores in liver and skeletal muscle, AMPK content was assessed in these tissues. Representative immunoblots and stain-free images of (**g**) liver and (**j**) skeletal muscle collected from male DKI and WT mice in the fed state. Quantified (**h**) AMPK α and (**i**) AMPK β1 in liver, and (**k**) AMPK α and (**l**) AMPK β2 in skeletal muscle are shown. Male mice, *n* = 6–9, 20–32 wk. $ *p* < 0.05, $$$ *p* < 0.001 versus fed condition from same genotype; * *p* < 0.05, ** *p* < 0.01, *** *p* < 0.001, **** *p* < 0.0001.

Given the role of AMPK in tissue glycogen storage and dynamics, we measured phosphorylation of AMPK and its downstream substrate acetyl-CoA carboxylase (ACC) in WT and DKI mice in the fed, overnight fasted, 5 h-post-glucose gavage, and 24 h-post-glucose gavage and refeeding states in liver (Figure 7a) and skeletal muscle (Figure 7d). In liver, there was an overall condition effect detected for AMPK α T172 phosphorylation relative to total protein. Specifically, DKI mice displayed increased phosphorylation in the overnight fasted and 5 h-post-glucose states compared to the fed state (Figure 7b). However, no individual genotypic differences were observed between WT and DKI mice (Figure 7b). There were also no differences detected in ACC S79 phosphorylation between WT and DKI liver observed in any condition (Figure 7c). In skeletal muscle, there was a trend for a genotypic effect for increased phosphorylation of AMPK α T172 in DKI mice compared to WT that did not reach statistical significance (*p* = 0.054), and post hoc analysis did not identify any significant differences in any individual condition (Figure 7e). There were also no differences observed in downstream ACC S79 phosphorylation between genotypes or across conditions (Figure 7f). 

### 2.8. DKI Mice Have Reduced AMPK Protein Content in Liver and Skeletal Muscle

Given the established roles for the AMPK α and β subunits in maintaining cellular energy balance and the observed increases in total body and fat mass as well as alterations in glycogen dynamics in DKI mice, the relative tissue protein content of these AMPK subunits was assessed in the fed state in both liver (Figure 7g) and skeletal muscle (Figure 7j). Compared to WT, liver from DKI mice displayed a ~56% reduction in total AMPK α protein content (Figure 7h). Moreover, DKI liver had a ~72% reduction in AMPK β1 protein content compared to WT (Figure 7i). In skeletal muscle from DKI mice, total AMPK α was reduced by 36% (Figure 7k) and total AMPK β2 was reduced by ~38% relative to WT (Figure 7l). However, decreased tissue AMPK content in DKI mice was not associated with any differences in *Prkab1* and *Prkab2* gene expression in liver and skeletal muscle, respectively, in the overnight fasted state (Figure S1). Finally, given the observed increase in whole-body fat mass and altered patterns of substrate utilization in DKI mice, AMPK protein content was also assessed in adipose tissue in the fed state. AMPK α, β1, and β2 content were reduced by ~44%, ~53%, and ~47%, respectively, in epididymal fat from DKI mice relative to WT (Figure S1).

## 3. Discussion

This study aimed to investigate the phenotypic effects of the DKI mutation used to chronically disrupt whole-body AMPK-glycogen binding in vivo on whole-body composition, glucose homeostasis, and tissue glycogen dynamics. To address this aim, we utilized a DKI mouse model in which whole-body glycogen binding was disrupted by simultaneously targeting both the AMPK β1 and β2 subunit isoforms. We report that DKI mutation of residues critical for AMPK-glycogen binding in both AMPK β subunit isoforms—AMPK β1 W100A and β2 W98A—leads to (1) increased body mass, associated with increased fat and lean mass; (2) hyperinsulinemia and glucose intolerance; (3) reduced fed liver glycogen levels and altered glycogen dynamics in skeletal muscle; and (4) reductions in AMPK protein content in the primary glycogen-storing tissues liver and skeletal muscle, as well as adipose tissue.

Recent research has identified isoform-specific phenotypic effects in single AMPK β subunit isoform KI mice, including whole-body glucose handling, tissue fat accumulation, and AMPK protein content and kinase activity [14]. The AMPK DKI mouse model generated for the present study displayed robust changes in body mass and body composition, as well as impaired glucose tolerance relative to WT mice when glycogen-binding capacity was disrupted in both β isoforms, similar to the whole-body phenotype observed in AMPK β2 W98A single KI mice. In the present study, we found increased rates of CHO oxidation in DKI mice compared to WT both in the fed, basal state and in response to overnight fasting, concomitant with reduced liver glycogen in the fed state and increased skeletal muscle glycogen depletion following an overnight fast in DKI mice. In response to overnight fasting-induced glycogen depletion, no changes in tissue AMPK content were observed in WT mice and DKI mice with chronic disruption of AMPK-glycogen binding. The lack of changes between tissue AMPK content in the fed state and following overnight fasting (~14 h) was consistent with previous findings showing no effects of fasting-induced energy stress and accompanying changes in glycogen content on liver and skeletal muscle AMPK content following prolonged 36 h fasting in mice [15] and 48 h fasting in humans [16], respectively. Despite the observed reductions in tissue AMPK content in DKI relative to WT mice, levels of phosphorylation of AMPK’s downstream substrate ACC detected in DKI liver and skeletal muscle were similar to WT in the fed, fasted, and refed states. These data are consistent with observations from AMPK β1 W100A and β2 W98A single KI mice [14] and suggest that either the remaining AMPK protein is sufficient to maintain downstream signaling to ACC, or the AMPK cellular pool that phosphorylates ACC is distinct from the pool lost with chronic disruption of glycogen binding. 

The DKI mutation resulted in increased body mass, whole-body lean and fat mass, decreased ambulatory activity, and the main effect for hyperphagia. Overall genotype effects for increased carbohydrate oxidation and decreased fat oxidation were observed in DKI mice. Furthermore, increased serum TAG levels in DKI compared to WT mice may suggest that the DKI mutation alters fat availability (i.e., lipolysis) and/or reduces tissue fat uptake. Despite leptin’s central role in regulating food intake [17] and increased circulating levels in obesity [18], there were no genotype differences detected in serum leptin concentration in either the fed or 6 h fasted states. Furthermore, we observed a reduction in AMPK protein content in adipose tissue in DKI mice compared to WT. While adipose is not a primary glycogen storing tissue, it does contain glycogen [19,20]; therefore, this may contribute to the observed reduction in AMPK content similar to that observed in liver and skeletal muscle. While the functional relevance of glycogen in adipose is currently unknown, increased levels of glycogen accumulation in adipose tissue have been associated with obesity [21,22]. AMPK is believed to play a role in regulating adipose metabolism and development [23]. Further research is warranted to elucidate the potential roles of AMPK-glycogen binding in the regulation of adipose tissue glycogen metabolism. 

AMPK functions as a central regulator of energy utilization and its dysfunction is associated with the onset of obesity, disruptions in glucose homeostasis, and the development of insulin resistance in insulin-sensitive tissues such as liver and skeletal muscle [24,25]. We therefore assessed glucose and insulin tolerance in DKI and WT mice. As hypothesized, DKI mice exhibited elevated blood glucose concentrations in response to IPGTT compared to WT. Glycogen stores, particularly in the liver, play an important role in maintaining euglycemia [15,26] and are critical for maintaining glucose tolerance [27]; therefore, the reduced hepatic glycogen levels observed in DKI mice may contribute to the whole-body glucose intolerance observed in these mice. DKI mice also displayed hyperinsulinemia in the 6 h fasted state, characteristic of the early stages of metabolic abnormalities observed in the progression of insulin resistance [28]. However, we did not observe any impairments in blood glucose responses during an insulin challenge indicating that DKI mice maintain whole-body insulin sensitivity. Additionally, there were no reductions in content nor phosphorylation status of glucose-transport related proteins in skeletal muscle in the fed state. Skeletal muscle is the primary tissue responsible for post prandial glucose disposal [29], suggesting that skeletal muscle insulin sensitivity is maintained in DKI mice despite hyperinsulinemia and whole-body glucose intolerance. 

Given the role of glycogen as an important source of glucose during energy stress, we examined the phenotypic effects of the DKI mutation on acute whole-body and tissue responses to fasting. At the whole-body level, DKI mice displayed reduced ambulatory activity and reduced rates of fat oxidation compared to WT during an overnight fast, consistent with observations in the fed state. These alterations in substrate utilization in response to fasting in DKI mice are consistent with reciprocal findings in mice with constitutively active liver AMPK α, which exhibited a more rapid shift to and higher reliance on fat utilization during fasting compared to WT [30]. In contrast, whole-body AMPK β1 knockout (KO) mice display a reduction in RER and increased rates of fat oxidation at the onset of fasting [31]. These disparate responses observed in transgenic AMPK models versus the DKI model in the present study suggest the presence of tissue-, subunit-, and/or isoform-specific roles for AMPK in regulating whole-body substrate utilization patterns in response to acute energy stress. 

In the fed state, DKI mice displayed reduced liver glycogen content, in line with findings in fed whole-body AMPK β2 KO mice, which also demonstrate reduced hepatic glycogen levels [32]. DKI mice showed no impairment in the ability to utilize liver glycogen during the overnight fast, as the absolute liver glycogen content following fasting was similar between genotypes. However, when accounting for the lower liver glycogen levels observed in the fed state in DKI mice, the overall glycogen utilization in DKI mice was significantly reduced relative to WT. At 24 h-post-glucose gavage and refeeding, DKI liver glycogen returned to levels observed prior to fasting. Liver glycogen levels can remain suppressed during resynthesis following fasting and exercise, given the greater dependence on liver glycogen to maintain euglycemia in rodents [33]. The maintenance of liver glycogen synthesis capacity in DKI mice is contrary to observations in liver-specific AMPK α KO mice, which had similar reductions in liver glycogen as WT mice following a 24 h fast, but attenuated resynthesis following 6 h of refeeding [26]. These results suggest that loss of liver AMPK α reduces glycogen deposition, leading to a limited capacity for glycogenolysis to maintain euglycemia in response to fasting. However, in the current study, DKI mice displayed elevated blood glucose levels and similar serum glucagon levels compared to WT following an overnight fast, as well as increased rates of CHO oxidation and intact signaling proteins underlying glucose uptake in skeletal muscle. These findings suggest that the levels of glucose release from the liver via glycogenolysis and/or gluconeogenesis likely remain intact in DKI mice during energy stress.

DKI mice had lower glycogen accumulation in liver compared to WT. This difference in the glycogen “ceiling” may be due to changes in glycogen particle structure in DKI mice; however, there were no genotypic differences in the content of enzymes associated with glycogen particle formation (i.e., GS, GBE, and glycogenin) or degradation (i.e., GP and GDE) in the livers from fed mice. Furthermore, there were no genotypic effects observed for changes in phosphorylation of GS S641 in the fed, fasted, or refed states. GS S641 is a target site of glycogen synthase kinase-3 [34,35] and dephosphorylation is associated with increased enzyme activity [36,37]. Similar levels of phosphorylation of AMPK T172 and GS S641 between genotypes suggest that glycogen resynthesis was not impaired in the DKI liver, therefore, we did not measure phosphorylation of other GS sites, such as the AMPK site GS S7 [38]. It is likely that the lower capacity to store glycogen observed in DKI mice was due to the increased reliance on CHO oxidation in the fed and fasting states compared to WT, limiting the accumulation of hepatic glycogen. Together, these findings suggest that the DKI mutation used to chronically disrupt AMPK-glycogen interactions may not impair glycogenolysis and glycogen repletion in liver but may be responsible for determining peak hepatic glycogen accumulation via altering substrate utilization.

Given the relatively low levels of glycogen in murine skeletal muscle, mice tend to preserve muscle glycogen stores even during extended periods of fasting [15]. However, considering the lower total availability of liver glycogen in DKI mice, and their increased reliance on CHO both at rest and during energy stress, it is possible that an energy challenge such as an overnight fast could result in DKI mice utilizing skeletal muscle glycogen while WT muscle glycogen levels remained unchanged. Therefore, it is plausible that due to an impaired ability to utilize fats and/or a preference for CHO substrates, DKI mice may use more skeletal muscle glycogen during fasting. Alternatively, given the elevated blood glucose concentrations observed in DKI mice compared to WT following overnight fasting, it is also possible that increased skeletal muscle glycogen degradation in DKI mice was due to impaired skeletal muscle uptake of circulating glucose, despite no observed changes in the glucose transport-related proteins in the fed state.

Glycogen resynthesis in skeletal muscle is a metabolic priority following energy stress, and full resynthesis typically occurs within 3–5 h of recovery and refeeding [39,40]. For example, resynthesis of skeletal muscle glycogen following exercise is promoted by the depletion-induced activation of GS and/or increased glucose uptake via exercise-induced translocation of GLUT4 to the plasma membrane [36,41,42]. Notably, in the current study, skeletal muscle glycogen levels in DKI mice remained suppressed compared to fed levels both 5 h-post-glucose gavage to promote glycogen resynthesis and following 24 h with refeeding. A recent study in inducible muscle-specific AMPK α KO mice found that loss of AMPK resulted in reduced glycogen resynthesis rates in skeletal muscle following exercise and glucose administration, leading to blunted glycogen repletion after 5 h of recovery [43], suggesting that skeletal muscle AMPK plays a crucial role in this process. Surprisingly, the inability to resynthesize skeletal muscle glycogen in DKI mice was not accompanied by any reductions in glycogen-associated proteins (i.e., GS, GBE, and glycogenin) or changes in phosphorylation of GS S641. Our findings suggest that disruption of AMPK’s glycogen binding capacity and/or the associated reductions in AMPK protein content in glycogen storing tissues lead to previously underappreciated consequences on regulation of glycogen dynamics, potentially through AMPK’s interactions with glycogen and/or indirectly by shifting substrate utilization patterns.

Previous studies involving cell-free assays [6] and acute glycogen-depleting exercise interventions performed in rodents [10] and humans [13,44] have demonstrated increased AMPK phosphorylation and kinase activity in association with reduced glycogen availability. These findings helped form the hypothesis that AMPK-glycogen binding may serve a role in sensing stored energy [6], whereby depletion of glycogen releases AMPK, allowing it to become activated and phosphorylate its downstream substrates to promote energy production. While these studies have helped establish the existence of this interaction in vitro as well as putative roles of these interactions in acute changes in glycogen availability in vivo, the physiological consequences of disrupting AMPK-glycogen binding in vivo remained to be elucidated. In the present study, we observed reductions in total AMPK α content in a range of metabolically active tissues and disruptions in metabolism associated with the DKI mutation. This is similar to observations in studies using tissue-specific [45] and isoform-specific [32,46,47] AMPK β KO models; however, in DKI tissues we do not observe a complete loss of AMPK protein content that is characteristic of KO models. We cannot distinguish whether the DKI phenotype and associated reductions in tissue AMPK content are due to disruption of AMPK-glycogen binding and/or due to AMPK destabilization independent of glycogen binding. As these phenotypic effects in DKI mice cannot be attributed directly or exclusively to AMPK-glycogen binding, the possibility of glycogen binding-independent AMPK destabilization warrants further research. It is well recognized that the β subunit plays an essential role in stabilizing the αβγ heterotrimer and the C-terminus (residues 186–270) of the β subunit is sufficient to anchor the α and γ subunits [3]. The C-terminus is structurally distal to the CBM (residues 68–163) responsible for glycogen binding. However, when considered along with findings that mutation of the CBM of the starch or glycogen binding proteins results in loss of protein and/or instability [48], our results suggest that the CBM and/or glycogen binding capacity of multiple proteins, including AMPK, may be involved in the regulation of protein content and/or stability. In contrast to reductions in expression of *Prkab1* in liver and *Prkab2* in skeletal muscle in AMPK β1 W100A and β2 W98A single KI mice versus WT in the fed state [14], we did not observe any changes in liver *Prkab1* or muscle *Prkab2* expression in overnight fasted DKI mice, suggesting that changes in gene expression do not contribute to reduced AMPK protein content. However, reduced tissue AMPK content in DKI mice is consistent with results from AMPK β1 W100A and β2 W98A KI models [14], suggesting that the β subunit CBM may play a role in maintaining pools of cellular AMPK protein and, therefore, metabolic homeostasis.

In summary, we demonstrate that the DKI mutation utilized to chronically disrupt whole-body AMPK-glycogen interactions in vivo leads to phenotypic effects including disruptions in whole-body metabolic homeostasis and tissue glycogen dynamics that are associated with reduced AMPK protein content in glycogen-storing tissues including liver, skeletal muscle, and adipose tissue. DKI mice also have increased body mass and adiposity, reduced rates of fat oxidation, as well as impaired glucose handling and hyperinsulinemia. Furthermore, DKI mice display increased rates of CHO oxidation in the fed state and during fasting-induced energy stress, associated with reduced basal liver glycogen levels and altered skeletal muscle glycogen utilization and repletion. The DKI mutation used to disrupt whole-body AMPK-glycogen binding in mice leads to simultaneous increases in serum insulin levels and adiposity, contributing to the progression of glucose intolerance characteristic of the early stages in the development of insulin resistance. Collectively, these findings highlight the roles of AMPK in maintaining energy balance, glucose homeostasis, and glycogen dynamics and suggest that AMPK-glycogen binding may serve potential physiological roles in cellular, glycogen-storing tissue, and whole-body energy homeostasis.

## 4. Materials and Methods

### 4.1. Animal Models

CRISPR/Cas9 gene targeting was used to generate two whole-body single KI mouse models on a C57BL/6J background. Tryptophan residues in the AMPK β subunit isoforms that are critical for glycogen binding [6,8]—W100 within the β1 isoform (W100A) and the analogous residue in the β2 isoform (W98A), respectively—were mutated to alanine, as described previously [14]. Heterozygous mice possessing either the β1 W100A or β2 W98A KI mutation were subsequently crossed to generate AMPK β1 (W100A)/β2 (W98A) DKI mice (Figure 1a) with chronic disruption of AMPK-glycogen binding. Homozygous carriers of the DKI mutations were used for breeding, and DKI and WT breeders were annually backcrossed to generate heterozygous mice and rederive the homozygous DKI line used for experimentation. Confirmatory genotyping was performed from tail samples by TransnetYX (Cordova, TN, USA).

Experiments were undertaken using male AMPK DKI and age-matched WT control mice. Only male mice were used to maintain adequate DKI female breeder availability and ensure sufficient age-matched DKI mice were readily available for analyses. Mice were group-housed in a temperature (22 °C) and humidity-controlled facility with 12:12 h light and dark cycle. Mice were given *ad libitum* access to a standard chow diet (6% fat, 20% protein and 29% starch; Barastoc, Ridley Agriproducts, Pakenham, Victoria, Australia) and water, unless otherwise specified. All mouse procedures were performed under approval of the St. Vincent’s Hospital (Melbourne, Victoria, Australia) Animal Ethics Committee (AEC 025-15 and 011-19), conforming to all the requirements of the National Health and Medical Research Council of Australia (NHMRC) and in accordance with the Australian code of practice for the care and use of animals for scientific purposes (8th Edition 2013). 

### 4.2. Whole-Body Composition Measurements

Whole-body composition (fat mass, lean mass, and water weight) was assessed by nuclear magnetic resonance using the EchoMRI Body Composition Analysis system (EchoMRI, Houston, TX, USA). Measurements were undertaken in the fed state (0900 h).

### 4.3. Metabolic Caging

Mice underwent metabolic cage analyses using the Comprehensive Lab Animal Monitoring System (CLAMS, Columbus Instruments, Columbus, OH, USA). Mice had either *ad libitum* access to chow or were fasted overnight. Mice with *ad libitum* access to chow were singly housed for ~60 h total at 21 °C and food intake, infrared-based ambulatory activity, O_2_ consumption (VO_2_), CO_2_ production (VCO_2_), and RER (VCO_2_·VO_2_^−1^) were measured continuously. The first ~6 h served as the acclimatization period and were not included in CLAMS data analyses. Following acclimatization, hourly, light/dark cycle and 24 h averages were calculated. TEE and rates of fat and CHO oxidation were calculated as previously described [49]. For responses to fasting, mice were acclimatized (~6 h) before food was removed at the start of the dark cycle (1800 h). Mice were fasted overnight (~14 h) and ambulatory activity, VO_2_, VCO_2_, and RER were measured continuously, and TEE and substrate oxidation rates were calculated. Mice were then returned to their home cages with *ad libitum* access to chow.

### 4.4. Intraperitoneal Glucose Tolerance and Insulin Tolerance Testing

Commencing at 0800 h, mice were fasted either 6 h prior to IPGTT or 4 h prior to IPITT, then injected with glucose (1 g·kg^−1^ total lean mass; Sigma-Aldrich, St. Louis, MO, USA) or insulin (0.5 U·kg^−1^ total body mass; Humulin, Eli Lilly and Company, Indianapolis, IN, USA), respectively. Blood glucose was monitored via tail tip bleed using a glucometer (Accu-Check Performa, Roche Diagnostics GmbH, Mannheim, Germany). Mice were allowed at least one week of recovery between tests.

### 4.5. Fasting and Refeeding Protocol

Mice underwent a fasting and refeeding protocol to assess glycogen depletion and repletion in response to energy stress and availability. A cohort of mice was subjected to an overnight fast (~14 h) while a control fed group had continued *ad libitum* access to food. The following morning shortly after the start of the light cycle (0800 h), blood glucose was measured in all mice via tail tip bleed using a glucometer. Fed mice and a subset of fasted mice were euthanized via cervical dislocation and tissues were collected and immediately snap-frozen for subsequent analysis. The remaining fasted mice received an oral gavage of glucose (3.6 g·kg^−1^ total body mass) and blood glucose responses were monitored over a period of 5 h, after which mice were either euthanized and tissues collected or were returned to their home cages with *ad libitum* access to food. Blood glucose was measured in the remaining mice 24 h after administration of the glucose gavage, and mice were then euthanized, and tissues were collected.

### 4.6. Serum Analysis

Blood samples collected retro-orbitally were left to clot for 30 min at room temperature in non-coated polypropylene tubes. Samples were then centrifuged at 10,000× *g* for 5 min at 4 °C, and the resulting serum supernatant was aliquoted and stored at −80 °C. Hormone and lipid concentrations were assessed in these aliquots using leptin (Abcam, Cambridge, UK), glucagon, and ultra-sensitive mouse insulin (Crystal Chem, Elk Grove Village, IL, USA) ELISA kits, and NEFA-C (Wako Pure Chemical Industries, Osaka, Japan) and TAG (Abcam) colorimetric assay kits.

### 4.7. Assessment of Tissue Glycogen Content

Snap-frozen gastrocnemius muscle (~40 mg) and liver (~25 mg) samples were chipped under liquid N_2_, freeze-dried overnight, powdered, and dissected free of visible blood and connective tissue. Aliquots of powdered tissue (~2–4 mg) were then alkaline extracted, and the supernatant was used for quantification of glycogen content using spectrophotometry as described previously [50]. Absorbance was measured at 340 nm using a SpectraMax Paradigm plate reader (Molecular Devices, San Jose, CA, USA) and data were acquired using SoftMax Pro microplate data acquisition software (Molecular Devices).

### 4.8. Immunoblotting 

Snap-frozen liver, gastrocnemius muscle, and adipose tissue samples were lysed in homogenization buffer containing 50 mM Tris. HCL, pH 7.5, 1 mM EDTA, 1 mM EGTA, 10% glycerol, 1% Triton-X (Sigma-Aldrich), 50 mM sodium fluoride, 5 mM sodium pyrophosphate with cOmplete Protease Inhibitor Cocktail and PhosSTOP phosphatase inhibitor tablets from Sigma-Aldrich. Lysed tissue samples were centrifuged at 16,000× *g* for 30 min at 4 °C, and supernatant protein content was determined using the bicinchoninic acid method (BCA; Pierce, Rockford, IL, USA). Tissue lysates (10 mg protein·well^−1^) were run on 4–15% or 4–20% precast stain free gels (Bio-Rad, Hercules, CA, USA) and transferred to PVDF membranes (Merck Millipore, Burlington, MA, USA). Membranes were blocked with 7.5% BSA in Tris-buffered saline containing 0.1% Tween 20 from Sigma-Aldrich (TBS-T) for 1 h at room temperature, then incubated with primary antibodies with rocking as detailed in Table 2. After washing with TBS-T, membranes were incubated for 1 h at room temperature in either an anti-rabbit IgG (GAR) or anti-mouse IgG (GAM) horseradish-peroxidase-conjugated secondary antibody from Cell Signaling Technology (CST; Danvers, MA, USA) in TBS-T. Proteins were detected via chemiluminescence using SuperSignal West Femto Maximum Sensitivity Substrate (Thermo Fisher Scientific, Waltham, MA, USA) and imaged using the ChemiDoc Imaging System (Bio-Rad). Band intensities of total protein content and protein phosphorylation were normalized to the total lane protein from the respective stain free image using Image Lab software (version 6, Bio-Rad) as described previously [51] and phosphorylation was expressed relative to respective total protein content. Primary and secondary antibody details are listed in Table 2.

### 4.9. RNA Extraction and Gene Expression Analysis

RNA from snap-frozen overnight fasted (~14 h) liver (15 mg) and skeletal muscle (25 mg) was extracted using TRIzol LS Reagent (Life Technologies, Carlsbad, CA, USA). RNA was treated with DNase I (Sigma-Aldrich) and reverse-transcribed using SuperScript VILO Master Mix (Life Technologies). Pre-Amplification was completed using TaqMan Custom PreAmp Pool and PreAmp Master Mix (Applied Biosystems, Foster City, CA, USA). Quantitative polymerase chain reaction (qPCR) was performed using a CFX Connect Real-Time System (Bio-Rad). Gene expression was normalized to GAPDH using TaqMan Fast Advanced Master Mix (Applied Biosystems). *Prkab1* (liver, AMPK β1; Mm01201921_m1), *Prkab2* (skeletal muscle; Mm01257133_m1), and *Gapdh* (liver and skeletal muscle; Mm99999915_g1) TaqMan Gene Expression Assays were used (Applied Biosystems). Relative levels of mRNA were assessed using the ΔΔCt method.

### 4.10. Statistical Analyses

Comparisons between genotypes were analyzed using two-tailed Student’s t testing (WT versus DKI) or two-way analysis of variance (ANOVA; WT versus DKI comparisons over time or condition). Measurements of body mass and composition were analyzed using linear mixed models. If significance was detected using ANOVA or linear mixed models, Bonferroni post hoc testing was applied where appropriate. All statistical analyses were performed using GraphPad Prism software (version 8, GraphPad Software, La Jolla, CA, USA). Significance was set at *p* < 0.05. All data are presented as mean ± standard error of the mean (SEM).

## Figures and Tables

**Table 1:** Tissue masses from male WT and AMPK DKI mice. Pancreas, heart, gastrocnemius muscle, and soleus muscle were collected from mice in the fed state and weighed.

Tissue	WT Tissue Mass (mg)	DKI Tissue Mass (mg)	*p*-Value
Pancreas	120 ± 12.0	167 ± 16.5 *	0.029
Heart	159 ± 10.3	176 ± 11.6	0.27
Gastrocnemius	306 ± 12.9	328 ± 17.1	0.11
Soleus	16 ± 1.8	14 ± 1.8	0.35

Data are presented as mean ± standard error of the mean (SEM); *n* = 8–14, 17–20 wk old mice. * *p* < 0.05.

**Table 2:** Antibodies and protocols used for immunoblotting.

Target	Primary Antibody	Incubation	Secondary Antibody
AMPK α p-T172	CST 2531	Overnight	GAR CST 7074
	1:1000	4 °C	1:5000
AMPK α	CST 2532	Overnight	GAR CST 7074
	1:1000	4 °C	1:5000
AMPK β1/β2	CST 4150	Overnight	GAR CST 7074
	1:1000	4 °C	1:5000
ACC p-S79	CST 11818	Overnight	GAR CST 7074
	1:1000	4 °C	1:5000
ACC	CST 3662	Overnight	GAR CST 7074
	1:1000	4 °C	1:5000
Akt p-S473	CST 9271	1.5 h	GAR CST 7074
	1:1000	Room temperature	1:5000
Akt	CST 4691	Overnight	GAR CST 7074
	1:1000	4 °C	1:5000
AS160 p-T642	CST 4288	Overnight	GAR CST 7074
	1:1000	4 °C	1:5000
AS160	Abcam ab24469	Overnight	GAR CST 7074
	1:1000	4 °C	1:5000
GLUT4	CST 2213	Overnight	GAM CST 7076
	1:1000	4 °C	1:2000
GS p-S641	CST 47043	Overnight	GAR CST 7074
	1:1000	4 °C	1:5000
GS	CST 3886	Overnight	GAR CST 7074
	1:1000	4 °C	1:5000
Glycogenin1	Abcam ab11171	Overnight	GAR CST 7074
	1:1000	4 °C	1:2000
GBE1	Abcam ab180596	Overnight	GAR CST 7074
	1:1000	4 °C	1:2000
GP	Antibody generated by B.E.K. laboratory	Overnight	GAR CST 7074
	1:1000	4 °C	1:2000
GDE	Antibody generated by B.E.K. laboratory	Overnight	GAR CST 7074
	1:1000	4 °C	1:2000

## Data Availability

The data that support the findings of this study are available from the corresponding author upon reasonable request.

## Supplementary Materials

The following are available online at https://www.mdpi.com/article/10.3390/ijms22179616/s1, Figure S1: DKI mice display no differences in liver *Prkab1* or muscle *Prkab2* gene expression versus WT, and DKI adipose tissue displays reduced AMPK content.
