# Supplementary material for: Mice with Whole-Body Disruption of AMPK-Glycogen Binding Have Increased Adiposity, Reduced Fat Oxidation and Altered Tissue Glycogen Dynamics
Source: Int J Mol Sci. 2021 Sep 5;22(17):9616. doi: 10.3390/ijms22179616 (PMC8431764; doi:10.3390/ijms22179616)
Supplement: Supplementary file 1 [file ijms-22-09616-s001.zip › ijms-1360055-supplementary.pdf]

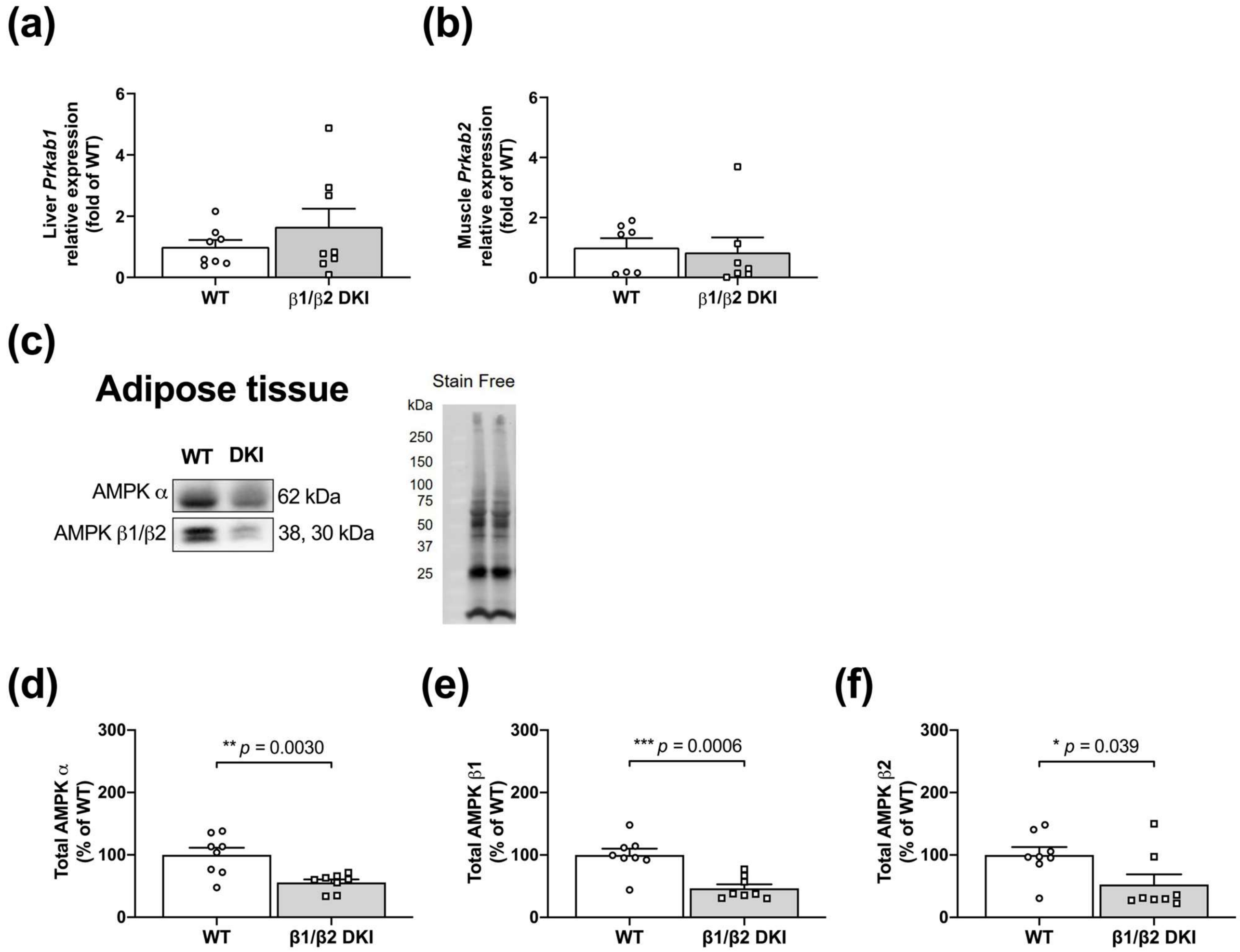

Figure S1. DKI mice display no differences in liver *Prkab1* or muscle *Prkab2* gene expression versus WT, and DKI adipose tissue displays reduced AMPK content. Gene expression was assessed in DKI and WT liver and skeletal muscle using qPCR. (**a**) Relative *Prkab1* (AMPK β1) gene expression in WT and DKI liver and (**b**) relative *Prkab2* (AMPK β2) gene expression in WT and DKI skeletal muscle. Overnight fasted (~14 h), male mice, n = 7–8, 20–32 wk. AMPK subunit protein content was assessed in epididymal adipose tissue. (**c**) Representative immunoblots and stain free images collected from fed DKI and WT mice. Quantified (**d**) AMPK α, (**e**) AMPK β1, and (**f**) AMPK β2. Male mice, n = 8, 17–20 wk. * $p < 0.05$, ** $p < 0.01$, *** $p < 0.001$, **** $p < 0.0001$.
